# The Relation of On-Ice and Off-Ice Performance at Two Different Performance Levels in Youth Ice-Hockey Players

**DOI:** 10.5114/jhk/187238

**Published:** 2024-05-17

**Authors:** Robert Roczniok, Petr Stastny, Dominik Novak, Lukas Opath, Artur Terbalyan, Martin Musalek

**Affiliations:** 1 Sports Science Institute, The Jerzy Kukuczka Academy of Physical Education in Katowice, Katowice, Poland.; 2 Faculty of Physical Education and Sport, Charles University, Prague, Czech Republic.; 3 Department of Physical Education and Sport, Faculty of Arts, Matej Bel University in Banska Bystrica, Banska Bystrica, Slovakia.

**Keywords:** testing, exercise, motor control, skills, condition, speed

## Abstract

Ice hockey requires two levels of specific agility, involving different abilities, where the level of agility and their constraints might vary by the performance level. Therefore, this study aimed to compare the relationship level between on-ice and off-ice change of directional speed (COD) of youth hockey players at two performance levels. The study was conducted during the hockey season, including U16 elite players (n = 40) and U16 sub-elite players (n = 23). Both groups performed specific on-ice fitness tests (4-m acceleration, 30-m sprint, and 6 x 54-m tests, an on-ice Illinois agility test with and without a puck) and off-ice tests consisting of non-arm swing countermovement jumps (CMJs), broad jumps, and pull-ups. Pearson correlation showed that the acceleration performance of elite players was related to the CMJ (r = −0.46) and the broad jump (r = −0.31). Sub-elite players showed stronger dependence of the 30-m sprint on the CMJ (r = −0.77) and the broad jump (r = −0.43), the relation of pulls ups (r = −0.62) and the CMJ (r = −0.50) to the 6 x 54-m test, yet no association to acceleration. Elite players differ between off-ice and on-ice performance constraints, where their skating sprint is less related to their vertical and horizontal take-off abilities than in sub-elite players. Sub-elite players’ off-ice power determines their sprint and repeated sprint performance. COD performance of elite and sub-elite players is based on different conditioning constraints.

## Introduction

Ice hockey is a team sport demanding athletes’ multi-dimensional physical and technical development (Burr et al., 2008; Vigh-Larsen et al., 2022), which requires two levels of specific agility including skating and puck control (Slavicek et al., 2022). Due to the intermittent nature of this discipline, it is highly dependent on the anaerobic energy supply (Vigh-Larsen et al., 2022) and characterized by repetitive intervals of high energy expenditure with shifts lasting 30–80 s including both high acceleration and velocity bouts (Cox et al., 1995; Douglas and Kenedy, 2020; Lau et al., 2001; Montgomery et al., 1988; Stanula and Roczniok, 2014). In order to compete at the elite level, players need to improve their fitness, including anaerobic endurance, strength, power, and aerobic endurance (31% aerobic metabolism) (Cox et al., 1995; Dominik et al., 2019; Glaister, 2005; Montgomery, 1988; Stanula et al., 2016; Roczniok et al., 2016). Moreover, explosive activities should go hand in hand with highly skilled technical actions and changes of directional speed (COD), which constitute the performance equilibrium that directly predicts the selected athlete's on-ice efficiency (Delisle-Houde et al., 2019; Nightingale et al., 2013; Peyer et al., 2011; Roczniok et al., 2016). It has been suggested that strength and conditioning coaches of ice hockey teams need to perform a comprehensive battery of tests during the pre-season fitness assessment (Delisle-Houde et al., 2019; Huard Pelletier et al., 2021), which include general and specific performance tests off- and on-ice (Daigle et al., 2022; Nightingale et al., 2013; Rago et al., 2022). However, the selection of test batteries is not typically related to players' performance levels.

The validity and use of physical testing have been controversial among practitioners and scientists. Some contend that only sport-specific tests are helpful (Nightingale et al., 2013; Runner et al., 2016), while others suggest that generic tests provide a valuable understanding of the underlying physical resources of performance factors (Mendez-Villanueva and Buchheit, 2013). However, elite-level ice hockey requires a high level of fitness in terms of power and high-intensity intermittent exercise capacity (Vigh-Larsen et al., 2019), where on-ice speed and endurance differ markedly between elite and sub-elite players. However, this difference is not in body composition or countermovement jump (CMJ) results (Vigh-Larsen et al., 2020). Thus, it raises the question of whether off-ice performance has a similar or a different relationship to on-ice performance at different players' performance levels.

Determining the weaknesses and vital parts of specific physical preparation of ice hockey players is essential for improving the training process, especially for youth players. In this regard, a helpful method is to compare athletes' specific and general fitness tests at different playing levels. The International Ice Hockey Federation (IIHF) world ranking reflects the performance level, where the IIHF provides a ladder system for its member national ice hockey teams. It is based on a system that awards points for each team's results in IIHF-sanctioned competitions over the previous four years. The ranking is used to determine seeding and qualifying for upcoming IIHF events.

Ice hockey requires two levels of specific agility, which are based on different abilities. The levels of agility and their constraints might vary by the performance level. Therefore, this study aimed to compare the level of the relationship between on-ice and off-ice change of directional speed (COD) of youth ice hockey players at two performance levels. We hypothesized that elite young hockey players would present a higher relationship between on-ice and off-ice performance.

## Methods

### Participants

The elite group consisted of 40 male members of the wide selection for the U16 national ice hockey team of the Czech Republic (aged 15.2 ± 0.3 kg, body height 177 ± 5.3 cm, body mass 69 ± 5.8 kg) and 23 athletes from the Polish U16 national team represented sub-elite players (aged 15.4 ± 0.3 kg, body height 181 ± 5.8 cm, body mass 71 ± 7.8 kg), goaltenders did not take part in on-ice testing. Participants were advised about the study's possible risks and benefits, and their consent to participate was received. Each participant was asked to get a whole night's sleep of at least 8 h before testing, to abstain from any ergogenic substances for 48 h before testing, and not to perform any physical activity that might affect their performance. All the athletes possessed up to date medical examinations confirming proper health status and the ability to perform high-intensity exercise. A signed informed consent form was also obtained from the parents of all players participating in this study.

### Study Design

The cross-sectional design evaluated the relationship between non-specific (off-ice) and specific (on-ice) performance. Participants performed the first off-ice tests after a 10-min warm-up, including vertical and horizontal jumping and shoulder mobility trials. Off-ice tests included CMJs without using arms, standing long jumps (broad jumps), and pull-ups on the bar with a pronated grip. On-ice testing started 60 min after finishing off-ice testing and began with a 10-min warm-up, including skating cross-overs, progressive accelerations, and skating agility. Specific fitness tests were performed on ice and included a 30-m maximum sprint with measurements at the 4^th^ and the 30^th^ m, the on ice Illinois agility test with and without a puck, and the 6 x 54-m repeated sprint test. The study protocol and the research were conducted following the guidelines of the Declaration of Helsinki (2013), and the research project was approved by the Ethics Committee for Scientific Research at the Charles University, Faculty of Physical Education and Sport (approval code: 144/2019; approval date: 21 May 2019).

### Measures

#### 4-m Acceleration and a 30-m Sprint

Both tests were completed within one acceleration of forward sprint skating, where photocells (TIMY3 timing device, ALGETIMING GmbH, Lustenau, Austria) were placed 4 m and 30 m from the goalie line. The first photocell on the goalie line recorded the start of the sprint, the second photocell recorded the 4-m acceleration, and the finish photocell the 30-m sprint (Novak et al., 2020; Stastny et al., 2023) on a straight sprint track along the barrier of an ice hockey rink. The starting photocell sensor was placed 30 cm above the ice, and both finishing sensors were placed 1.2 m above the ice. Players were instructed to put an ice hockey stick across the starting line during the initial sprint position and began testing from their preferred knee flexion. The better result out of the two trials was used for further statistical analyses.

#### Modified Illinois Agility

**Figure 1:**
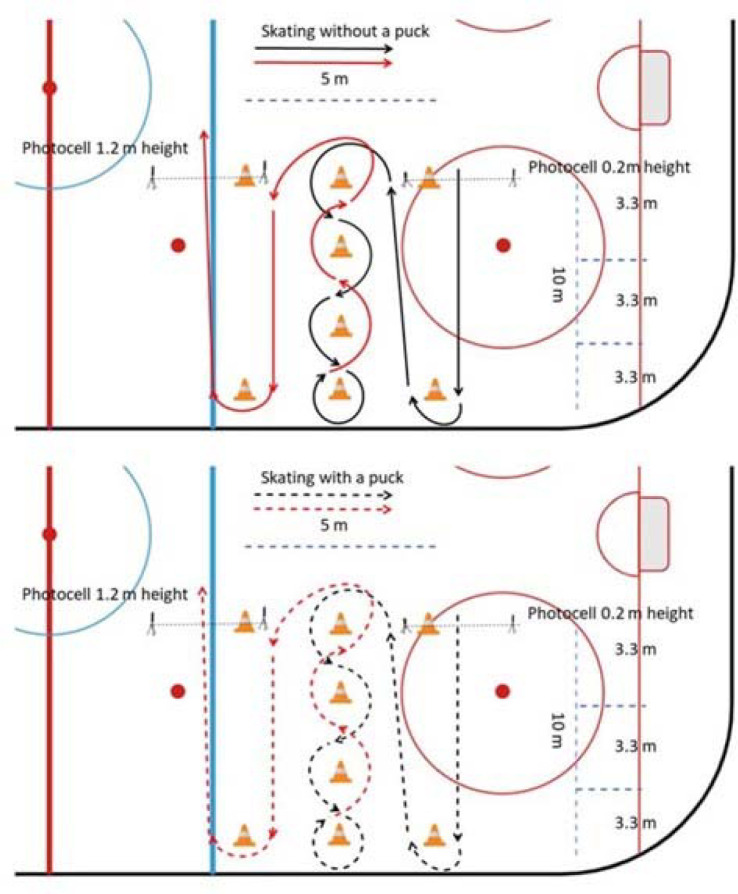
The Illinois agility test performed with and without a puck.

Modified Illinois agility tests were administered on-ice on a regular ice hockey rink. Players skated the Illinois track under two conditions with and without a puck. This COD test was selected because it provides acceptable validity and reliability (standard error of measurement of 0.07, 95% confidence interval of 0.20 for minimal detectable change) for equipment control (Makhlouf et al., 2022). Moreover, this test showed a relationship between off-ice COD and anthropometry of youth ice hockey players (Slavicek et al., 2022). The initial starting position was with the ice hockey stick across the 30-cm high photocell line and athletes’ feet behind it; a 1.2-m high photocell marked the finish line. Players skated first without a puck (Figure 1A) and then with a puck (Figure 1B), where the stick handling during the 60-m Illinois COD track was allowed. Players had to skate the entire track with both feet, and a stick had to control the puck throughout the whole track. Two successful trials of each Illinois test were collected, and better results were used for statistical analysis. Differences between time without a puck and with a puck were expressed as percentage differences. Players performed 2–4 trials of each test variation until they completed two successful trials, with the better result used in further analyses.

#### 6 x 54-m Repeated Sprint Test

The 6 x 54-m repeated skating sprint test was selected to estimate anaerobic capacity on-ice. Players skated with an ice hockey stick all-out diagonally across the ice hockey rink for 54 m, where they stopped by a side brake and returned to the starting position (second 54 m). Thus, players skated six 54 m shifts with a brake and no rest interval in between. Photocells were set up similarly to skating speed testing. Photocells collected data including all 54-m raw results, which were used for statistical analyses and total skating time. This test was performed only once because of its physical demands and was also used to determine the sprint speed for 50 m, using an additional photocell on the track. Players were instructed to perform each shift at maximum intensity without spreading their effort throughout the test.

#### Skating Efficiency Index

The skating efficiency index (SSI) was measured during the 6 x 54-m repeated sprint test, where the number of strides was counted for the 324-m track. The number of strides was divided by the skating speed across the whole test (strides/m/s)^1^ (Allisse et al., 2019). A lower value in the SSI is translated into better skating efficiency based on fewer strides to reach and maintain skating velocity. Skating efficiency is typically related to a player's skating technique and glide ability.

#### Countermovement Jump

The CMJ was performed using two three-axis piezoelectric force plates (Kistler, Winterthur, Switzerland). For the CMJ, participants started standing comfortably with their hands on their hips. Then they squatted by bending their knees, hips, and ankles on cue and then jumped as high as possible (Burr et al., 2007). Players performed four separate jumps with 2–5-s rest intervals, and the best result considering jump height was used for further analyses.

#### Broad Jump

The broad jump (a bilateral standing long jump) was performed from a pelvis-wide standing position, performing a squat and subsequent horizontal jump, where countermovement was permitted. Players jumped three times with a 3–5- min rest interval between subsequent trials, where they attempted to jump as far as possible. The longest jump distance was used for statistical analyses. This procedure provides acceptable reliability, with a reported intertrial difference of 0.3 ± 12.9 cm (Ortega et al., 2008) and an intraclass correlation of 0.97 (Rahman et al., 2021). Broad jump length was determined by distance from the starting line at the tip of the feet and the heel of the foot, which landed closer to the starting line. Players had to land bilaterally to the squat position.

#### Pull-Ups

A standard procedure was applied during pull-ups, which were performed on a gymnastic bar with a diameter of 2.8 cm and a length of 240 cm. This test has high reliability confirmed by the intra-class correlation coefficient of 0.96–0.99 and 3% of the smallest worthwhile change (Coyne, 2015). Players had to use a pronated grip with the hands placed at shoulder width. Each repetition started from a full hang position with elbows fully extended, shoulders fully flexed, and the shoulder girdle elevated. Hips and knees were either in a neutral position or self-selected flexion. The instructors checked the correct initial body position. At the instructor's command, players performed the pull-up explosively without swinging the trunk and lower limbs or kicking the legs. The upward phase ended when the participant’s chin passed the pull-up bar while maintaining a neutral head position, preventing the chin from being lifted to complete a repetition. After the concentric phase, the eccentric phase was performed by lowering the trunk back to the initial position at a self-selected speed with a maximum of 2-s rest intervals between repetitions. Pull-ups were performed as a single trial to failure.

### Statistical Analysis

The statistical analysis included basic descriptive statistics in the form of location, asymmetry variability, and concentration measures. The normality of distribution was verified using the Shapiro-Wilk test. The Student's *t*-test for means was used to check performance differences between the elite and sub-elite groups, and the Pearson correlation coefficient was used to determine the relationship between off-ice and on-ice performance. The correlation was interpreted following guidelines by Cohen (1988) as small (r = 0.1–0.29), medium/moderate (r = 0.3–0.49), or large (r > 0.5), whereas effect sizes in *t*-test were interpreted as small (*d* = 0.2–0.49), medium (*d* = 0.5–0.79), or large (*d* > 0.8). All analyses were performed using the Statistica v.13.1 package and an Excel spreadsheet (Microsoft Excel 2021 v. 16.0, USA) at a significance level of 0.05.

## Results

Analysis of the results began with the calculation of basic descriptive statistics, where no data disrupted the normality of distribution. Both groups were similar in age, body height, and body (Table 1), while in the 4-m sprint, 30-m sprint puck control, and the broad jump (Table 1), the effect sizes suggested moderate differences (Table 1). The elite group outperformed sub-elite players and obtained better results in the 50-m track and Illinois tests, the 6 x 54-m repeated sprint, the skating efficiency index, the CMJ, and pull-ups (Table 1).

**Table 1:** Performance and group differences in off-ice and on-ice-tests.

Variables	Group	Mean ± SD	±95% CI	*t*−test *p*		Cohen *d*	Mean difference raw (%)	Mean difference ±95% CI
Body height (cm)	Elite	177 ± 5.3	176–179		0.06	0.71	2.77 (1.53)	−0.17–5.7
	Sub-Elite	181 ± 5.8	178–183					
Body mass (kg)	Elite	69 ± 5.8	66.9–70.4		0.17	0.28		
	Sub-Elite	71 ± 7.9	67.6–74.5				2.35 (3.34)	−1.02–5.74
Age (years)	Elite	15.2 ± 0.3	15.1–15.3		0.91	0.66	0.01 (0.01)	
	Sub-Elite	15.4 ± 0.3	15.2–15.4					0.01–0.02
4 m (s)	Elite	0.868 ± 0.100	0.836–0.900		0.06	0.50	0.047 (5.2)	−0.003–0.096
	Sub-Elite	0.914 ± 0.083	0.879–0.951					
30 m (s)	Elite	4.295 ± 0.705	4.073–4.518		**<0.01**	0.47	0.732 (16.6)	0.616–0.848
	Sub-Elite	4.539 ± 0.183	4.460–4.618					
50 m (s)	Elite	7.039 ± 0.331	6.933–7.145		**0.01**	0.65	0.262 (3.6)	0.061–0.463
	Sub-Elite	7.300 ± 0.463	7.100–7.501					
Illinois with a puck (s)	Elite	16.1 ± 0.62	15.867–16.259		**0.01**	0.70	0.397 (2.4)	0.094–0.700
	Sub-Elite	16.5 ± 0.51	16.237–16.815					
Illinois without a puck (s)	Elite	15.3 ± 0.41	15.131–15.396		**<0.01**	0.95	0.480 (3.1)	0.261–0.699
	Sub-Elite	15.7 ± 0.43	15.560–15.928					
Illinois difference (%)	Elite	4.9 ± 2.4	4.1–5.7		0.34	0.22	0.600 (13.0)	−0.66–1.87
	Sub-Elite	4.3 ± 2.5	3.2–5.4					
6 x 54-m repeated sprint (s)	Elite	53.5 ± 2.3	52.79–54.29		**<0.01**	1.12	2.675 (4.9)	1.418–3.932
	Sub-Elite	56.2 ± 2.5	55.1–57.3					
Skating efficiency index (stride/m/s)	Elite	20.8 ± 1.9	20.2–21.4		**<0.01**	1.24		
	Sub-Elite	23.3 ± 2.1	22.4–24.2				2.52 (11.4)	1.49–3.55
Countermovement jump	Elite	37.9 ± 4.0	36.7–39.2		**<0.01**	1.43	5.90 (16.7)	3.81–7.99
(cm)	Sub-Elite	32.1 ± 4.1	30.3–33.9					
Broad jump (cm)	Elite	232 ± 10.1	228–237		0.08	0.48		
	Sub-Elite	223 ± 24.4	213–234				−8.79 (3.9)	−0.92–18.50
Pull-ups (n)	Elite	10.1 ± 3.7	8.9–11.2		**<0.01**	1.15	−4.18 (52.2)	−2.29–−6.07
	Sub-Elite	5.9 ± 3.6	4.4–7.5					

SD = standard deviation, CI = 95% confidence interval, **Bold** value marks significant differences by the *t*-test

Correlation analyses showed that jump results moderately correlated with acceleration and the 30-m sprint, yet with no other relation to skating variables in the elite group. The sub-elite group showed a moderate correlation among acceleration, 50-m sprint, 6 x 54-m repeated sprint, and jump results as well as a high correlation between the CMJ and the 30-m sprint (Table 2). Moreover, the elite group showed a moderate correlation between the skating efficiency index and 4-m and 30-m sprints (r = 0.41 r = 0.44, respectively).

**Table 2:** Relationship between on-ice and off-ice tests in elite and sub-elite U16 ice hockey players.

Measure	Group	4 m (s)	30 m (s)	50 m (s)	Illinois with a puck (s)	Illinois without a puck (s)	Puck control deficit (%)	6 x 54 (s)	SSI (stride/m/s)
Body height	Elite	−0.03	0.03	0.09	0.12	0.01	0.14	0.07	−0.08
	Sub-elite	0.27	0.30	0.12	−0.10	0.13	−0.22	−0.16	−0.03
Body mass	Elite	−0.08	−0.21	−0.17	0.07	−0.03	0.08	−0.29	−0.01
	Sub-elite	0.11	−0.09	0.02	−0.10	−0.02	−0.04	−0.30	0.19
Broad jump	Elite	**−0.32**	**−0.31**	0.08	0.05	−0.13	0.20	−0.05	0.05
	Sub-elite	**−0.41**	**−0.43**	−0.15	−0.23	−0.06	−0.14	−0.06	**−0.23**
CMJ	Elite	**−0.46**	**−0.46**	−0.18	−0.23	−0.22	−0.07	−0.11	0.01
	Sub-elite	**−0.34**	**−0.77**	**−0.49**	0.03	−0.32	0.29	**−0.50**	**−0.38**
Pull-ups	Elite	−0.10	−0.30	−0.15	−0.25	−0.10	−0.23	−0.06	−0.09
	Sub-elite	−0.20	−0.30	**−0.38**	−0.07	−0.13	0.04	**−0.62**	−0.10

Values are expressed in Pearson correlation r. Bold value is statistically significant at p < 0.05. CMJ = Countermovement jump

## Discussion

The present study aimed to compare the relationships between on-ice and off-ice COD of youth hockey players at two performance levels. We hypothesized that elite youth hockey players would present a higher relationship between on-ice and off-ice performance. Yet, this hypothesis must be rejected based on the results that showed the opposite effect. Sub-elite players had a higher relationship between off-ice and on-ice measurements, although the elite group achieved better on-ice and off-ice results. This effect might be explained by a higher level of skating technique in elite players represented by a lower skating efficiency index. The possible contribution of skating technique differences is supported by previous studies (Budarick et al., 2020; Shell et al., 2017), where elite players benefited from a higher functional range of motion and higher power transfer to a horizontal direction.

A previous study comparing two national U20 teams of Denmark and Finland (Vigh-Larsen et al., 2020) found a similar relationship between jumps and sprints (r = 0.40–0.47). In a similar way, the U20 Finland team outperformed Denmark U20 players in most off-ice and on-ice tests. On the other hand, another study found a strong CMJ correlation between ice sprint (r = 0.69) and agility performance (r = 0.51) (Vigh-Larsen et al., 2019), while our study observed such a correlation only in the sub-elite group between the CMJ and the 30-m sprint. Considering that elite players had better off-ice power and a better SSI, as well as that all sub-elite players’ sprints (short, long, and repeated sprint) were related to off-ice power, it seems that sub-elite players use their strength and power to a greater extent to reach maximum performance on ice without a precise skating technique. This may cause a greater tendency for fatigue and increased energy demands during repeated sprinting (Allisse et al., 2019), which again causes lower performance in repeated sprinting.

The test battery in our investigation was generic. It included metrics of speed, strength, power, and endurance. The tests were set up in this way to ensure that many players could complete all tests at the exact location on the same day each year. Similar assessments are commonly used by practitioners (Ebben et al., 2004) and scientists (Nightingale et al., 2014). Skating performance testing is essential for identifying specific performance of ice hockey athletes (Wagner et al., 2021). The battery of tests included on-ice trials we had used in previous studies (Roczniok et al., 2012) and reviewed quite recently (Stastny et al., 2023). Considering differences in agility, skating, and off-ice power, we can confirm that our testing battery provides sufficient sensitivity to evaluate different performance levels, with the most conspicuous result in this study being the mean and the minimal number of pull-ups performed by the sub-elite group (Table 1). The high pull-up performance differences could be partially explained by the bivariate relationship between upper-body strength and points scored in a game (Kniffin et al., 2017).

The general fitness score is highly interlinked with specific fitness and can influence it significantly, while specific test results are highest at a similar age (Wagner et al., 2021). Few studies have been conducted to investigate the association between jump tests and on-ice skating speed (Behm et al., 2005; Gupta et al., 2023; Mascaro et al., 1992), where vertical jump (r = 0.68−0.85) (Gupta et. al., 2023; Mascaro et al., 1992) and broad jump (r = 0.75) (Boucher et al., 2020) power were a precise predictor of on-ice skating speed. Moreover, changes in the broad jump correlate (r = 0.49) with skating improvement (Haukali et al., 2016) and determine the shot differential (r = −0.53) and average shift length (r = −0.49) (Delisle-Houde et al., 2018). Furthermore, the 40-yard sprint times combined with squat jump, drop jump, and leg press measures, as well as flexibility are the most efficient predictors of on-ice linear speed (R^2^ = 0.516) (Behm et al., 2005). Our results showed relatively lower correlations in the elite group, while some were relatively high, like r = 0.7 (Table 1) in the sub-elite group, which underlines the possible effect of skating technique. On the other hand, a study by Boland et al. (2019) did not show any relationships between the CMJ and on-ice skating or game performance, yet their study sample consisted of female participants.

This study found many differences between elite and sub-elite players, which should be reduced to improve performance of sub-elite players. There is some evidence that high-intensity aspects of specific on-ice performance (repeated sprints) can be improved in a short period of time, such as four weeks, even in youth national team ice hockey players (Jeppesen et al., 2019). Therefore, the speed endurance training method can be helpful for sub-elite players. Throughout the year, National Hockey League (NHL) players attend 2–4 weekly off-ice fitness workouts (Ebben et al., 2004); thus, strength and conditioning coaches of sub-elite teams need to consider similar fitness programming to adapt athletes to elite-level competition demands.

The main limitation of this study consists in the high heterogeneity of test batteries used in the ice hockey literature (Quinney et al., 2008). Future research should apply similar test batteries. For example, the NHL combine tests used in several studies (Burr et al., 2008; Burr et al., 2007; Vescovi et al., 2006). Such an approach provides worthwhile fitness measures specific to ice hockey or information on players' potential future success. To evaluate an athlete's on-ice playing potential, the NHL combines different testing methodologies which include a number of off-ice assessments. The Wingate 30-s anaerobic power test, a cycle ergometer VO_2max_ test, muscular endurance tests (push-ups and sit-ups), grip strength testing, bench presses, standing long jumps, and vertical jumps are among the tests carried out during the annual evaluations (Hajek et al., 2020). The NHL tests do not contain specific performance metrics; thus, the overall skating performance test for ice hockey (SOSPT) may be a good option for assessing specific skills. A prior study (Vescovi et al., 2006) verified the SOSPT and found it reliable and valid for determining specific overall skating performance in ice hockey players.

## Conclusions

These findings made it possible to accurately assess the differences between youth elite and sub-elite national team players. Sub-elite players need more general fitness and skating technique, as their lack decreases specific on ice performance. Therefore, sub-elite hockey players in younger age categories need to improve their skating technique and significantly enhance their basic conditioning abilities. General conditioning variables are essential differentiators, even amongst elite athletes. Elite players differ in the relationship between off-ice and on-ice performance constraints, as their sprint is less related to vertical and horizontal take-off abilities than in sub-elite players. Sub-elite players' off-ice power determines their sprint and repeated sprint performance. COD performance of elite and sub-elite players is based on different conditioning constraints.
